# Update on Transcatheter Tricuspid Valve Replacement Therapies

**DOI:** 10.3389/fcvm.2021.619558

**Published:** 2021-02-15

**Authors:** Ythan H. Goldberg, Edwin Ho, Mei Chau, Azeem Latib

**Affiliations:** Albert Einstein College of Medicine/Montefiore Medical Center, Bronx, NY, United States

**Keywords:** tricuspid regurgitation, transcatheter valve implantation, structural valvular heart disease, tricuspid valve replacement, experimental devices

## Abstract

Severe tricuspid regurgitation is relatively common, especially in the elderly, and portends poor survival. Neither medical therapy nor conventional surgery is efficacious for most patients. In contrast, transcatheter tricuspid valve interventions are showing promise to improve quality of life and mortality. Although there is more clinical experience with transcatheter tricuspid valve repair, there are many patients for which repair is either not possible or cannot optimally reduce the severity of tricuspid regurgitation. Transcatheter tricuspid valve replacement is rapidly emerging and may ultimately become the preferred treatment option. In this review, we discuss transcatheter tricuspid valve replacement, analyze the devices in development and in clinical trials, and highlight the advantages and drawbacks of transcatheter tricuspid valve replacement vs. repair.

## Introduction

Clinically significant tricuspid regurgitation (TR) is quite common, with at least moderate TR occurring in >1 in 200 of the general population and 4% of those ≥75 years of age (1). Five year survival is <30% with increased hospitalization for heart failure and atrial fibrillation leading to a high burden on the health care system. Moderate or severe TR is associated with higher mortality independent of left ventricular ejection fraction or pulmonary artery systolic pressure, and among patients with heart failure, severe TR is associated with a mortality rate 2.5 times that of those with no TR (2, 3).

Over 90% of the time, TR is functional with normal anatomic leaflets and subvalvular apparatus (1, 4). In these cases, tricuspid annular dilatation and/or leaflet tethering develops as a result of dilatation of the right atrium, right ventricle, or both (5). The underlying etiology is most commonly pulmonary hypertension, either from left-sided heart failure, mitral or aortic valve disease, or primary pulmonary causes. Atrial fibrillation may be both a marker of disease progression as well as a cause of annular dilatation due to atrial remodeling (6, 7).

Since 1967 when Braunwald et al. (8) published outcomes showing improvement in TR after mitral valve replacement, the pre-dominant approach to severe functional TR has been conservative management. There were studies at the time, however, which suggested a benefit to corrective surgery (9, 10). Currently, tricuspid valve surgery for functional TR is recommended only when performing surgery for concomitant left-sided valve disease. Mortality for isolated tricuspid valve surgery carries an in-hospital morality of 8.8%, with surgical replacement carrying a risk nearly twice that of repair (11).

There now exist options, albeit mostly experimental, for transcatheter tricuspid valve repair (TTVr). Unfortunately, many patients who would benefit from repair do not have suitable valve anatomy, usually die to large coaptation gaps, short and/or extremely tethered septal leaflets, inadequate imaging or just the overall complexity of the right-sided atrioventricular valve that may have four or more leaflets in up to 40% of cases (12). Transcatheter tricuspid valve replacement (TTVR) has the potential to fulfill this unmet clinical need. There are two broad categories of TTVR-orthotopic, where the valve is deployed at the tricuspid valve annulus, and heterotopic, where valves are deployed in one or both vena cavae. This review will describe each type of valve, along with its potential advantages, disadvantages, and the status of their clinical trials.

## Orthotopic Transcatheter Tricuspid Valves

### Patient Selection for Transcatheter Tricuspid Valve Replacement vs. Transcatheter Tricuspid Valve Repair

When considering whether repair or replacement is the better strategy there are several factors to consider. A large coaptation gap >6–8 mm and non-central regurgitant jets are associated with poor procedural success (13). The presence of calcification in the potential grasping target and immobile or severely retracted leaflets (especially the septal leaflet) with extensive tenting distance are unlikely to have good outcomes with repair. If residual tricuspid regurgitation is expected to be moderate or worse after repair, TTVR may be more appropriate. On the other hand, complete elimination of TR may lead to afterload mismatch and worsening of RV failure, which will be discussed later.

One common exclusion for TTVr is the presence of a permanent ventricular pacing lead that interacts with the tricuspid leaflet. Prospective data on the effect of lead extraction on TR severity is limited and could result in worsening severity, but may be considered for patients without leaflet trauma (14). In contrast, leads that are entrapped during TTVR usually (but not always) avoid damage (15). Tricuspid valve stenosis is an exclusion for TTVr because any repair strategy will inevitably reduce valve area and increase the gradient. Certain congenital conditions such as Ebstein anomaly, as well as primary leaflet abnormalities due to endocarditis, inflammatory diseases, or iatrogenic causes are also not suitable for TTVr.

The largest valve available in active clinical trials currently in the U.S. is the 52 mm EVOQUE valve. While larger valves are in development and may be incorporated into trials, individuals with a very large annulus should be considered either for TTVr, or a heterotopic valve implanted in the SVC, IVC, or both. Eccentric annuli may be prone to significant paravalvular leaks. If the right ventricle is not large enough it may not be able to accommodate the delivery system. Other geometric factors such as the height, position and angle of the IVC to the tricuspid annulus may make positioning of the transcatheter valve difficult or impossible. A high-quality transesophageal echocardiogram and gated cardiac CT scan are required to make accurate measurements. Pre-procedural planning also requires measurement of the inferior vena cava and iliac veins to ensure they are large enough to accommodate the delivery catheter, which is larger with a TTVR device compared with repair.

In patients at high risk for bleeding, a repair strategy may be preferable because lifelong anticoagulation is generally recommended after TTVR. Although there is no long-term data on the long-term durability of repair or replacement device, one advantage of transcatheter valves is the potential to perform valve-in-valve TTVR at a future time if needed.

### Potential Complications of Transcatheter Tricuspid Valve Replacement

Due to the relative immaturity of the field of TTVR, the full scope of complications is not fully known. However, all devices share certain risks and precautions that are worth noting. Regardless of the valve's anchoring mechanism, improper anchoring may lead to device malfunction, paravalvular leak, valve embolism, or valve thrombosis. Conduction abnormalities related to pressure or stretching that a transcatheter valve can place on the His bundle occur more frequently than with surgical or transcatheter repair. Because blood flow velocity is lower on the right side than on the left side of the heart, the risk of valvular thrombus formation is thought to be higher. Lifelong anticoagulation has been recommended, though switching to dual antiplatelet agents after 6 months has been proposed when there is no concomitant indication for long term anticoagulation.

Perhaps the most feared result following TTVR is its effect on RV systolic function and pulmonary hemodynamics. An acute increase in afterload on the RV following the elimination of TR may lead to worsening of right heart failure, or at least a failure to improve outcomes (16, 17). Whereas the RV is resilient in the setting of primary volume overload states, it is more sensitive to pressure overload. Post-procedure RV failure is more likely when the etiology of TR is functional in the setting of severe pulmonary hypertension, though the overall risk is likely to be lower than with open heart surgery (18). In addition to accurate determination of the etiology of tricuspid regurgitation, fundamental to patient selection and prognosis is a thorough evaluation of baseline RV function by echocardiography. This is more challenging than assessment of LV function assessment due to its asymmetric geometry, and several measurement techniques have been devised. These include tricuspid annular plane systolic excursion, tissue Doppler, RV end diastolic volume index, fractional area change, RV myocardial performance index, and global longitudinal strain. Treadmill or bicycle exercise echocardiography can be considered to assess for RV contractile reserve.

### Cardiovalve

**Figure 1:**
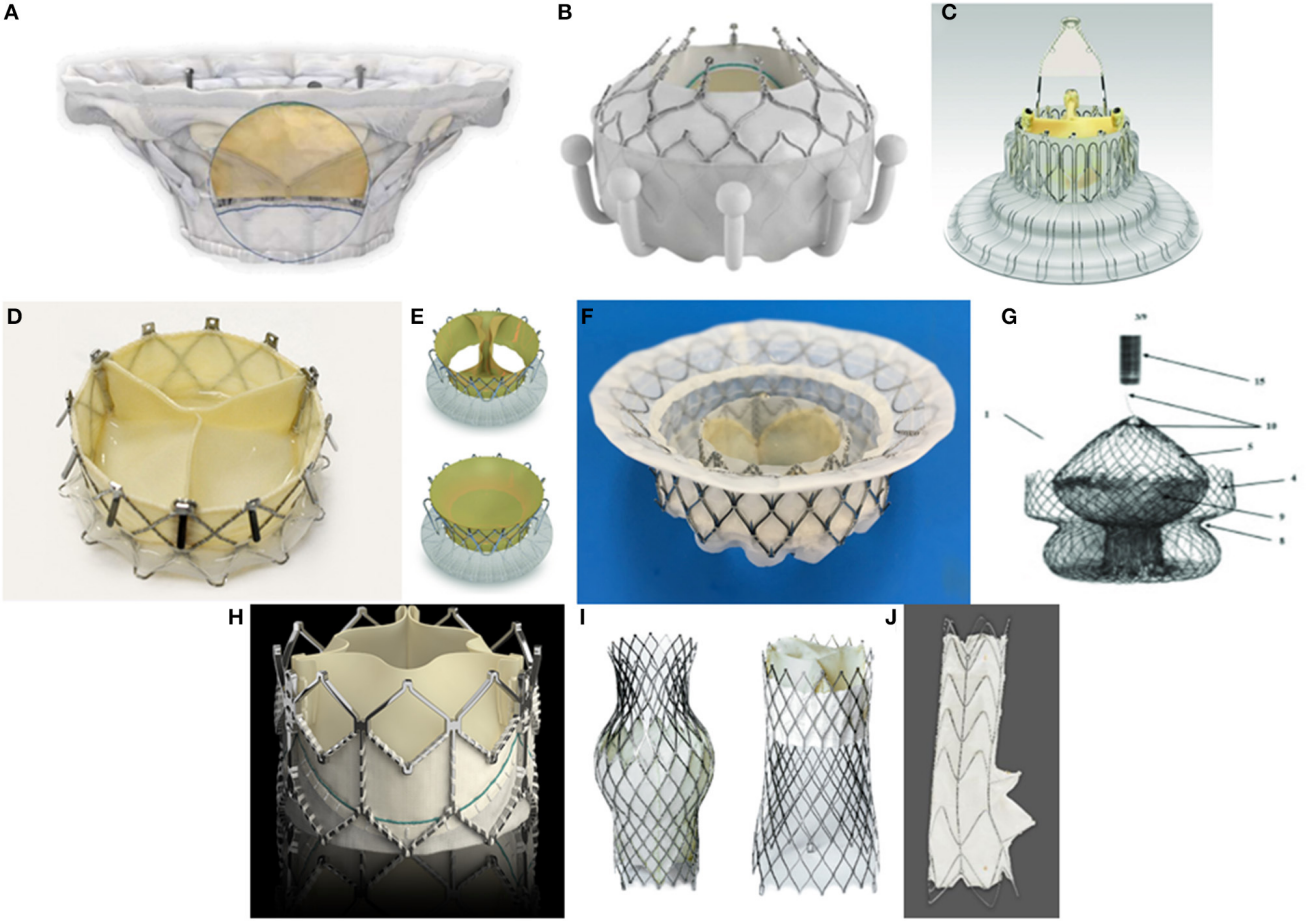
Orthotopic transcatheter valves: **(A)** Cardiovalve *(Boston Medical, Shrewsbury, MA, USA)*. **(B)** Evoque *(Edwards Lifescience, Irvine, CA, USA)*. **(C)** LUX-Valve *(Jenscare Biotechnology, Ningbo, China)*. **(D)** NaviGate *(NaviGate Cardiac Structures Inc., Lake Forest, CA, USA)*. **(E)** Trisol *(Trisol Medical, Yokneam, Israel)*. **(F)** Intrepid *(Medtronic Plc, Minneapolis, MN, USA)*. **(G)** Tricares *(TRiCares SAS, Paris, France)*. Heterotopic transcatheter valves: **(H)** Sapien XT *(Edwards Lifescience, Irvine, CA, USA)*. **(I)**. TricValve *(P*+*F Products* + *Features, Vienna, Austria)* **(J)**. Tricento *(New Valve Technology, Hechingen, Germany)*.

Cardiovalve (Boston Medical, Shrewsbury, MA, USA, Figure 1A) is composed of bovine pericardial leaflets mounted on a nitinol frame. Anchoring is achieved via leaflet grasping and an atrial flange and assisted by a proprietary anchoring and sealing element. Sizes are available in 5 mm increments from 45 to 55 mm, with a 60 mm valve in production. Access is obtained transfemorally with a 28Fr delivery system.

An early feasibility trial with Cardiovalve is currently underway in the U.S. (NCT04100720). Fifteen patients will be enrolled, and primary endpoints include intraprocedural technical success as well as significant device-related adverse events at 30 days.

### Evoque

The Evoque tricuspid valve replacement system (Edwards Lifescience, Irvine, CA, USA, Figure 1B) resembles its mitral valve counterpart, comprised of bovine pericardial leaflets with an intra-annular sealing skirt and anchors (Table 1). It is available in 44 and 48 mm sizes. A particularly notable advantage of Evoque is its low profile 28Fr delivery system which is employed via transfemoral access and the multiplanar steerable delivery system allows for coaxial deployment of the valve in most anatomies. While the Evoque mitral valve is in the early feasibility trial stage, the first in-human Evoque TTVR was only recently performed, albeit with excellent results at 6 month follow-up (19). Recently, data on 25 compassionate use cases was presented with a very high technical success rate of 92%, effective in reducing TR (88% with 0 or 1+ residual TR) and an excellent safety profile with no procedural deaths and 8% of patients requiring a permanent pacemaker (20).

**Table 1:** List of transcatheter tricuspid valve devices currently in development and testing.

**Device**	**Manufacturer**	**Composition**	**Anchor design features**	**Sizes (mm)**	**Access**	**Published data**	**Registered clinical trials**	**Patients**	**Primary endpoints**
**Orthotopic**									
Cardiovalve	Boston Medical	Nitinol frame, bovine pericardial leaflets	Leaflet grasping and atrial flange delivery	3: M/45, L/50, XL/55 (XL <60 in development)	28Fr transfemoral		Early Feasibility Study of the Cardiovalve System for Tricuspid Regurgitation (NCT04100720)	15 single arm	Intra-procedural technical success, SADE at 30 days
Evoque	Edwards	Bovine pericardial leaflets	Intra-annular sealing skirt and anchors	2 (44, 48)	28Fr transfemoral	1st in-human performed Mar 2020	N/A (mitral valve only)		
Lux-Valve	Jenscare Biotechnology	Self-expanding bovine pericardial tissue valve on nitinol stent covered by layer of polyethylene terephthalate	Leaflet fixation: 2 anterior leaflet clampers; septal anchoring: via interventricular anchorage	Annular 3 (50, 60, 70); leaflet inner diameter 2 (26, 28)	Transatrial insertion via minimally invasive right thoracotamy	35 patients, compassionate use	N/A		
NaviGate	NaviGate Cardiac Structures	Tapered nitinol stent with xenogenic pericardial leaflets	Atrial winglets, ventricular graspers	4 (36–52); oversize 2–5%	42Fr introducer via transjugular or transatrial (R thoracotomy)	32 patients, compassionate use	N/A		
TriSol	TriSol Medical	Self-expanding conical nitinol stent, porcine pericardium ventricular and polyester atrial skirts; single bovine pericardial dome shaped leaflet attached in 2 opposite central commisures (bileaflet)	Axial force; retrievable, repositionable		30Fr transjugular		N/A		
Intrepid	Medtronic	Dual stent system with 29mm bovine pericardial valve	Recoverable any time before final release	43, 46, 50	35 Fr transfemoral (29Fr in development)		N/A (mitral valve only)		
TRiCares	TriCares SAS	Self-exapanding bovine pericardial valve mounted on nitinol stent frame					N/A		
**Heterotopic**									
Sapien XT	Edwards	Balloon-expandable, cobalt-chromium frame, trileaflet bovine pericardial tissue valve, and polyethylene terephthalate (PET) fabric skirt	Requires preceding stent implantation	20, 23, 26, 29	14Fr or 16Fr	TRICAVAL ended prematurely due to high rate of dislodgement (not published)	HOVER (NCT02339974)	15 Single Arm	Procedural success: device success and no device/procedure related SAEs (30 days), patient success: no right heart failure related hospitatlization or advanced therapies; improvement in KCCQ, 6MWT or peak VO_2_
TricValve	P + F Products + Features	Self-expanding pericardial tissue on nitinol stents	N/A	SVC- 30 with variable hip protrusion up to 45; IVC- up to 43	27Fr transfemoral	First in-human	TRICUS (NCT03723239)	10 Single Arm	Major SAE (30 days), change in NYHA class (6 months)
							TRICUS Euro (NCT04141137)	35 single arm	Major SAE, KCCQ (30 days), KCCQ (3 months)
Tricento	New Valve Technology	13.5cm covered stent with landing zones in SVC and IVC and low intra-atrial porcine bicupsid valve segment. Short non-covered segment for hepatic vein inflow	N/A	Custom made up to 48	24Fr Transfemoral	First in-human	N/A		

### Lux-Valve

Lux-Valve (Jenscare Biotechnology, Ningbo, China, Figure 1C) is a self-expanding bovine pericardial tissue valve mounted on a nitinol stent covered by a layer of polyethylene terephthalate. Two anterior leaflet clampers attach to the native valve and an interventricular anchor attaches to the septum. As a result, the Lux-Valve does not rely on radial forces in order to secure its position. There are separate sizes for the annulus (50, 60, 70 mm) and the inner valve (26, 28 mm). The 32Fr system is placed into the right atrium through a minimally invasive right thoracotomy. Lux-Valve has been used in China on a compassionate-use basis, with plans to conduct an early feasibility trial in Canada underway (PMID 32646711)In 35 patients treated, implantation success rate was 100%, and all but two (5.7%) were alive at 30 days. RV volume, 6 min walk distance, and NYHA class were all significantly improved (21).

### The GATE System

The NaviGate transcatheter heart valve (THV) (NaviGate Cardiac Structures Inc., Lake Forest, CA, USA, Figure 1D) is a nitinol self-expanding tapered stent with a trileaflet equine pericardial valve. It is anchored with 12 tynes on the ventricular side to grasp native leaflets and 12 atrial winglets on the atrial side. Woven polyester microfiber over the atrial winglets helps to prevent compression of the conduction system. Access with a 42Fr introducer is obtained either through the internal jugular vein, or directly into the right atrium via thoracotomy. However, the jugular approach has been abandoned due complications related the extremely large sheath size and impossibility to achieve coaxiality with the relatively unsophisticated delivery system. Four sizes are available ranging from 40 to 52 mm, typically chosen with 2–5% oversizing in mind. Implantation feasibility was demonstrated in a pre-clinical model and a first-in-man procedure performed in a failed tricuspid annuloplasty ring via right anterolateral mini-thoracotomy (22, 23). Device durability and safety was demonstrated out to 4 months (24). NaviGate has been implanted on a compassionate use basis in Europe, the United States, and South America. In one series of five patients at a single institution with multiple severe comorbidities, technical success was achieved in all cases, there was one in-hospital death, and the others survived out to 30 days (25). In a report of 32 patients receiving NaviGATE on a compassionate use basis, implantation success was 100%, with all experiencing a ≥2 grade reduction in TR severity grade, and 30 day mortality was 12.5% (26).

### Trisol Valve

The Trisol valve (Trisol Medical, Yokneam, Israel, Figure 1E) is comprised of a self-expanding conical nitinol stent with a single bovine pericardial dome-shaped leaflet attached in two opposite central commissures for a bileaflet anatomical effect. It was designed to have a high closing volume in order to reduce the acute increase in afterload associated with elimination of severe tricuspid regurgitation. The valve is anchored via axial force applied to porcine pericardium ventricular and polyester atrial skirts. It is both retrievable and repositionable, and access is obtained via a 30Fr transjugular delivery system. The Trisol valve has demonstrated procedural feasibility and safety in a pre-clinical animal model.

### Intrepid

Intrepid (Medtronic Plc, Minneapolis, MN, USA, Figure 1F) is a dual-stent system with a 29 mm bovine pericardial valve. It is available in three sizes (43, 46, 50 mm). Transfemoral access is currently obtained via a 35Fr delivery system, though a 29Fr system is in development. An early feasibility trial with Intrepid for TTVR is about to start in the US and three compassionate use cases have been successfully performed (27).

### TRICares

The TRiCares (TRiCares SAS, Paris, France, Figure 1G) is a bovine pericardial self-expanding valve mounted on a nitinol frame. Though still early in its development, it received 2020 grant funding from the European Innovation Council to bring its product into the clinical realm.

### Which Valve to Consider

In order to determine which valve is most appropriate for an individual, certain characteristics such as annular size, angle of the inferior vena cava, leaflet anatomy, and femoral access should be considered. One commonly encountered problem with severe functional TR is large annular size. In this situation the Cardiovalve and EVOQUE valves are most likely to be successful. Intrepid, Cardiovalve, and EVOQUE all have dedicated delivery systems with advanced steering, which may be necessary when anatomy is less than ideal. Intrepid does not require leaflet capture in order to be deployed. EVOQUE has the smallest sheath size at 28Fr.

## Heterotopic Transcatheter Tricuspid Valves

### Patient Selection for Orthotopic vs. Heterotopic Transcatheter Tricuspid Valve Replacement

There are inevitably patients for whom a transcatheter valve is not an option, either due to geometric characteristics that preclude successful valve deployment, or right ventricular, hemodynamic, or clinical characteristics that suggest a low likelihood of benefit. Caval valve implantation may reduce venous regurgitation and improve right heart hemodynamics, ameliorating the effects of severe right heart failure such as anasarca, ascites, hepatic congestion and dysfunction, and exertional dyspnea to some degree (28). However, this procedure results in ventricularization of the RA, is unlikely to have any positive impact on RV function and remodeling, and should be considered a palliative procedure. It has been even more challenging than orthotopic TTVR because of the large and volume dependent size of the IVC and SVC, the risk of embolization, the risk of thrombosis and the risk of occluding the hepatic veins.

### Sapien

The Sapien line of balloon-expandible valves (Edwards Lifescience, Irvine, CA, USA, Figure 1H) is widely known for its FDA-approved indication for severe aortic stenosis, but has been used off-label to treat severe refractory TR. This trileaflet bovine pericardial valve is attached to a balloon-expandable cobalt-chromium frame with a polyethylene terephthalate skirt. Anchoring is only obtained by deploying a stent in the IVC as a landing zone prior to valve placement because the IVC diameter is usually too large for the 29 mm Sapien valve Care needs to be taken not to occlude the hepatic vein with the covered portion of the Sapien valve.

The first in-human Sapien caval implantation for severe refractory TR was reported in 2013 (29). Since then, Sapien valves has been used on a compassionate use basis with good success (30). However, the TRICAVAL trial to assess safety and efficacy of Sapien XT implantation in the IVC was ended prematurely due to a high rate of valve dislodgement (four out of 14 patients) (31). The HOVER trial is now ongoing and will evaluate safety, efficacy, and quality of life measures following IVC implantation of the Sapien XT (32).

### TricValve

TricValve transcatheter bicaval valves (P+F Products + Features, Vienna, Austria, Figure 1I) is a system of self-expanding bovine pericardial tissue valves mounted on nitinol stents. The SVC valve has a long skirt designed to minimize paravalvular leak, while the IVC valve has a short skirt in order to prevent occlusion of hepatic vein flow. A 24Fr transfemoral delivery system is used to implant the valves. TricValve is available in sizes of 25 and 29 mm for the SVC valve, and 31 and 35 mm for the IVC valve.

The first in-human TricValve system was successfully implanted, with improved symptoms at 8 weeks and 12 month follow-up (33, 34). TRICUS (NCT03723239), an early feasibility study of 10 patients undergoing heterotopic TTVR with the TricValve system is ongoing in the United States. Primary endpoints include serious adverse events at 30 days and change in NYHA class at 6 months. A parallel European study comprising 35 patients, TRICUS Euro (NCT04141137), is also underway.

### TriCento

The TriCento bioprosthesis (New Valve Technology, Hechingen, Germany, Figure 1J) is a 13.5 cm covered stent with landing zones in the SVC and IVC, and a low intra-atrial porcine bicuspid valve segment. There is also a short non-covered segment to allow for hepatic vein outflow. The stent is custom made based on pre-procedure imaging. Access is transfemoral via a 24Fr sheath.

The first in-human implantation in 2017 reported successful device function and reduced caval vein regurgitant volume after 3 months (35, 36). Since then a total of 31 TriCento bioprostheses have been implanted in Europe (37).

## Conclusion

Severe tricuspid regurgitation is no longer thought of as merely a marker of disease but is now widely thought of as a significant contributor to cardiac morbidity and mortality. With the rapid development of transcatheter tricuspid valve therapies, tricuspid regurgitation can now be corrected without incurring the untoward risks of conventional surgery.

TTVR devices have an advantage in many ways over both surgical TV replacement and transcatheter TV repair. They are less dependent on leaflet morphology or etiology of TR than repair devices, and without surgical morbidity and mortality risks associated with open heart surgery. As has been witnessed with TAVR, one may expect outcomes to only improve as each generation of device can address flaws in its predecessor, and as operators gain more experience performing the procedure. Patient selection is always paramount, and proper multimodality imaging with computed tomography, transthoracic and transesophageal echocardiography is crucial for deciding which procedure and which device is most appropriate for the individual patient.

Although transcatheter TV repair devices are closer to regulatory approval in the United States, replacement devices are actively being studied in the clinical arena. Early feasibility trials for TTVR are beginning or already underway for Evoque, Cardiovalve, Intrepid, and TricValve. TTVR is an emerging therapy for patients with severe functional tricuspid regurgitation who are not candidates for transcatheter repair or surgical replacement and would otherwise have an extremely poor prognosis. Much still needs to be learned about optimal device and patient selection, but at long last there is hope for those suffering from tricuspid regurgitation and without any other treatment options.

## Author Contributions

YG and AL: conception and design or analysis and interpretation of data, or both. YG, EH, MC, and AL: drafting of the manuscript or revising it critically for important intellectual content. YG and AL: final approval of the submitted manuscript. All authors contributed to the article and approved the submitted version.

## Conflict of Interest

AL is a consultant and advisory board member for Edwards Lifesciences, Abbott, Medtronic, and V-Dyne. The remaining authors declare that the research was conducted in the absence of any commercial or financial relationships that could be construed as a potential conflict of interest.
